# Effect of circuit structure on odor representation in the insect olfactory system

**DOI:** 10.1523/ENEURO.0130-19.2020

**Published:** 2020-05-15

**Authors:** Adithya Rajagopalan, Collins Assisi

**Affiliations:** 1 Janelia Research Campus, Howard Hughes Medical Institute, Ashburn, United States; 2 Division of Biology, Indian Institute of Science Education and Research, Pune, India

## Abstract

In Neuroscience, the structure of a circuit has often been used to intuit function – an inversion of Louis Kahn's famous dictum, `Form follows function' (Kristan and Katz 2006). However, different brain networks may utilize different network architectures to solve the same problem. The olfactory circuits of two insects, the Locust, *Schistocerca americana,* and the fruit fly, *Drosophila melanogaster*, serve the same function – to identify and discriminate odors. The neural circuitry that achieves this shows marked structural differences. Projection neurons (PN) in the antennal lobe (AL) innervate Kenyon cells (KC) of the mushroom body (MB). In locust, each KC receives inputs from 50% PNs, a scheme that maximizes the difference between inputs to any two of ~50,000 KCs. In contrast, in drosophila, this number is only 5% and appears sub-optimal. Using a computational model of the olfactory system, we show the activity of KCs is sufficiently high-dimensional that it can separate similar odors regardless of the divergence of PN-KC connections. However, when temporal patterning encodes odor attributes, dense connectivity outperforms sparse connections.

Increased separability comes at the cost of reliability. The disadvantage of sparse connectivity can be mitigated by incorporating other aspects of circuit architecture seen in drosophila. Our simulations predict that drosophila and locust circuits lie at different ends of a continuum where the drosophila gives up on the ability to resolve similar odors to generalize across varying environments, while the locust separates odor representations but risks misclassifying noisy variants of the same odor.

## Introduction

Neural circuits encode a variety of stimuli and perform a wide range of computations. The structure of the neural circuit (i.e., the organization and statistics of the connectivity between neurons in the circuit) plays a key role in restricting the kinds of computations that the circuit can perform (Marr 1969, Albus 1971, Hopfield and Tank 1986).

Understanding what different structural organizations imply for circuit function is an integral step towards generating a complete picture of brain function. These structure-function relationships are of particular interest in circuits that are trying to accomplish the same overarching goal while making use of different structural parameters. What advantages do the different parameter regimes provide in such situations? One such instance that has been explored recently, (Jortner, Farivar, and Laurent 2007, Jortner 2013, Litwin-kumar et al. 2017) is the functional effect of different densities of connections across species in the antennal lobe - mushroom body circuit of the insect olfactory system.

The insect olfactory system is arguably one of the most well-characterized neural circuits.

**Figure 1:**
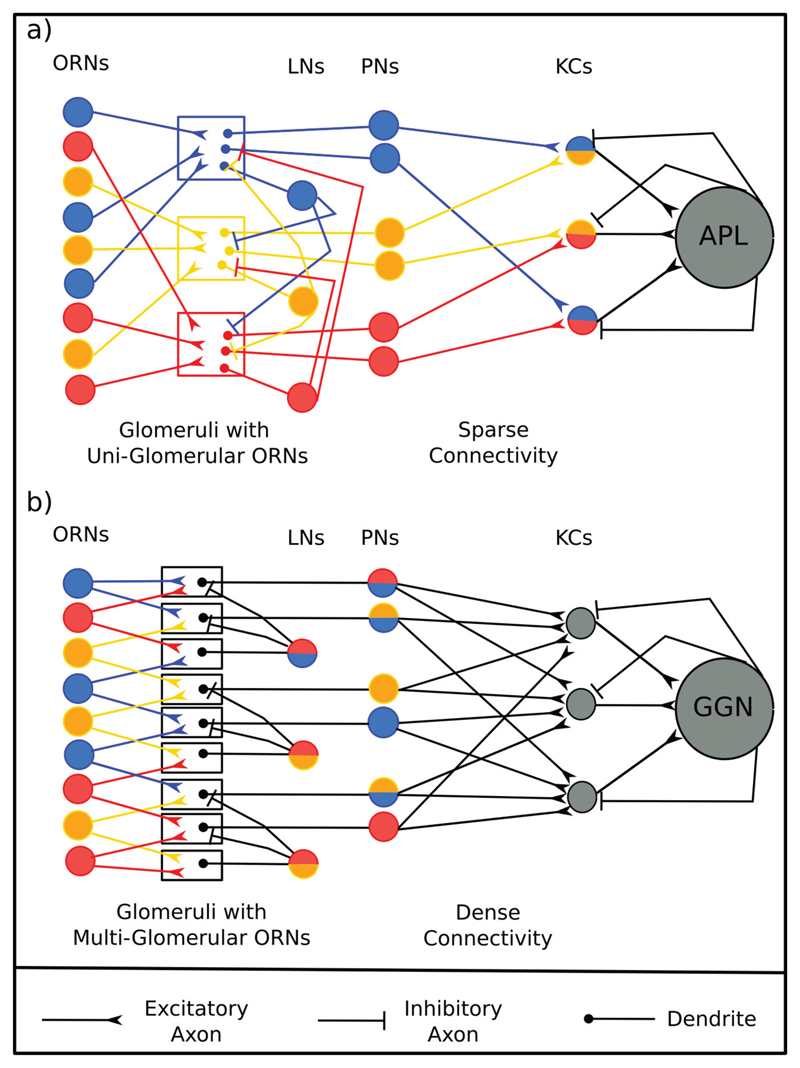
A schematic of the insect olfactory system A schematic of the olfactory system contrasting the structural parameters of the circuit in a)Drosophila melanogaster and b) Schistocerca americana.

Its compactness and simplicity, combined with the powerful genetic tools available, have allowed a detailed understanding of its structure and function. The circuit begins at the olfactory sensory neurons (OSNs) that convert odorant information from the environment into electrical signals that are passed on to higher brain regions (Hallem and Carlson 2004, 2006; Fisek 2014). The second level of the circuit is the Antennal Lobe (AL), where the principal excitatory neurons - Projection Neurons (PNs) - represent odors as dense spatiotemporal firing patterns (Laurent 1996b; Wehr and Laurent 1996; Wilson and Laurent 2005). The AL then feeds information to the Mushroom Body (MB), where Kenyon Cells (KCs) represent the odor as a spatially and temporally sparse pattern of firing (Javier Perez-Orive et al. 2002; Turner, Bazhenov, and Laurent 2008). A high spiking threshold and inhibitory inputs to KCs from a pair of large GABAergic neurons (Papadopoulou et al. 2011; Masuda-Nakagawa et al. 2014, Lin et al. 2014) maintains the sparseness of KC responses. The inhibitory GABAergic neurons are graded neurons whose membrane voltage is mediated by the activity of the KCs, thus forming a feedback inhibition loop [Figure 1]. Synapses immediately downstream of the KCs are plastic and thought to be the primary locus of associative memory in the insect (Heisenberg 2003, Hige, Aso, Modi, et al. 2015). KCs converge on to the Mushroom Body Output Neurons (MBONs). From the MBONs onwards, neuronal activity is related more with behavioral output than with stimulus representation (Aso et al. 2014; Hige, Aso, Rubin, et al. 2015). While the overarching goal of the MB circuit - to distinctly represent odors so as to facilitate learning and appropriate behavioral responses - appears to be conserved across species, the number of connections received from the AL to a given KC varies significantly. In the fruit fly, a sparse ~5% of all PNs synapse onto each KC, whereas in the locust, this number is dense (~50%) [Figure 1] (Caron et al. 2013; Jortner, Farviar and Laurent 2007). 50% connectivity seen in the locust olfactory system is thought to maximize the differences between the inputs received by individual KCs (Jortner, Farivar, and Laurent 2007; Jortner 2013). 5% connectivity observed in drosophila, must then make it a sub-optimal classifier. The combinatorial arguments that have been posited thus far do not consider the full spatiotemporal extent of an odor-evoked pattern of activity in the antennal lobe. To understand the implications of these contrasting connectivities, we tested the response of the fly and the locust olfactory networks to two different kinds of inputs – one, where odors were represented as spatiotemporal patterns of activity by AL neurons and another, where odors were represented only by the identity of active PNs. We show that an identity code allows a broad range of connection densities, including those seen in both the fly and locust, to distinguish different odors. However, with temporal variations, denser connectivities between PNs and KCs maximize the distance between odor representations. The sensitivity of the locust olfactory system, due to its dense connectivity, comes at a cost. Under changing environmental conditions, the same odor may generate different representations in PN space that the locust could potentially misclassify as distinct odors. Such misclassifications are less likely in the drosophila circuit where PN-KC connections are sparse. To elucidate the logic behind these connectivities, we simulated the distinct architectures of each insect. In drosophila, all the sensory neurons expressing a particular receptor type synapse onto PNs in a spatially circumscribed structure called a glomerulus. Sister PNs, that receive inputs from ORNs at a particular glomerulus, tend to fire in a highly correlated manner (Kazama and Wilson 2009) (though this is not the case in related mammalian cells –(Dhawale et al. 2010) where the activity, though correlated is different). In contrast, locust glomeruli receive input from multiple ORN types. We show that the glomerular architecture of the fruit fly improves the ability of the network to distinguish odors despite a low probability of PN-KC connections. Our simulations predict that the fruit fly and locust circuits lie at different ends of a continuum where the fruit fly gives up on resolution in odor space so that it can generalize across varying environments. This implies that very similar odors may be misclassified as the same odor as they are too similar to be resolved. The locust, on the other hand, maximally separates odor representations but runs the risk of misclassifying the same odor under different conditions.

## Methods

### Temporally patterned odor representations in AL circuits

**Figure 2:**
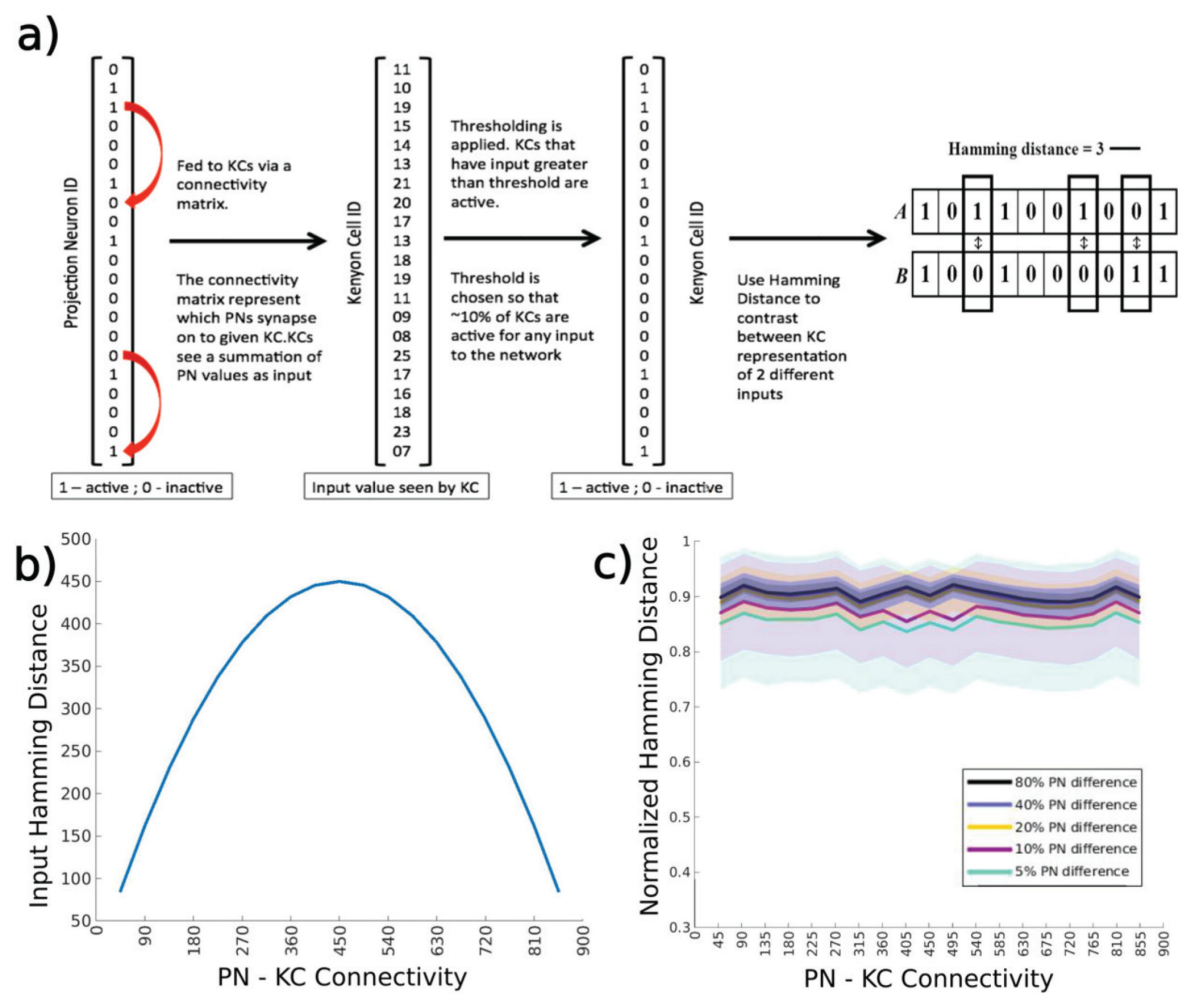
50% connectivity does not maximally separate KC representations when PN inputs are static a) The threshold model of KCs. The left-most vector represents the PN activity. This is combined through a connectivity matrix to give the input seen by each KC (a 50000-element long vector). Thresholding is then applied to define spiking KCs. b) The Hamming distance between inputs seen by two KCs is calculated for all possible pairs and averaged and plotted as a function of the PN-KC connectivity. c) The mean (± standard deviation) normalized Hamming distance between the activity of KC networks driven by two different inputs is plotted on the y-axis as a function of the PN-KC connectivity. Different shades plot the distance between odor representations that differed in 5-80% of the active PNs.

**Figure 3:**
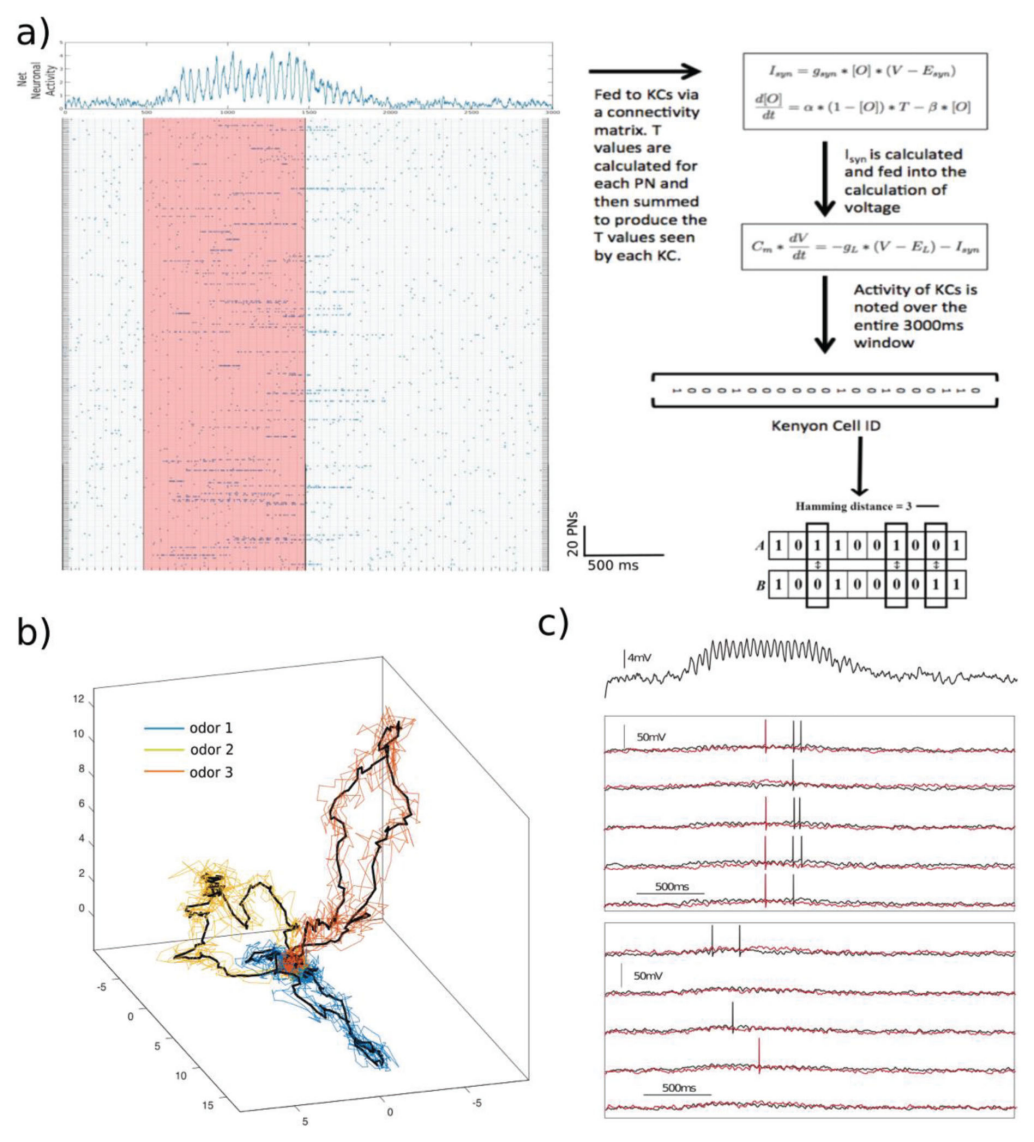
Simulation of temporally patterned PN inputs to a KC network. a) The matrix on the left represents the activity of a set of 900 PNs. Each row shows the activity of a single PN during a 3000ms time period. Blue dots show the time of a spike. The red region represents the time during which the odor was presented. On top, a summation of the activity of the entire PN network is shown clearly indicating the oscillations in the net PN activity. This input was used to calculate *T* and *^I^_syn_* (the synaptic input to KCs). The differences between the population representation of two inputs were calculated using the Hamming distance. b) Mean population response of 900 PNs projected onto the first three principal components for three odors is shown by the black traces. Individual trials are shown by the colored traces c) The mean membrane potential of all KCs shows a 20Hz oscillation. Bottom panels show the response of two KCs (in red and black traces) to two different odors. Only the first odor evokes a consistent response from this particular KC across 5 odor trials (middle panel). The second odor does not lead to reliable spiking in this example KC.

We modeled the odor representation in the AL in two ways. First, as a static representation consisting of a binary vector of length 900 (number of model PNs). Each element of the vector indicated only whether a particular PN was active (if the value at that position was 1) or not (0) [Figure 2a]. The second representation incorporated the temporal evolution of the odor. In the locust AL, odors elicit a temporal pattern of activity in PNs that begins with the onset of the odor. In experimental recordings, not all PNs show an odor specific response that begins immediately upon odor onset. Several neurons show increased activity many milliseconds after odor onset. Some PNs can show complex responses such as an increased level of activity to both odor onset and offset. However, it is likely that the onset and offset responses are largely seen in nonoverlapping groups of PNs (Saha et al. 2017). Here, we simulated PN spiking activity as continuous bursts. The spatiotemporal pattern generated by the PN population was defined by the onset, offset, and duration of PN bursts. Another important aspect to consider was the presence of oscillations in the Local Field Potential (LFP) in the 20-30Hz frequency range (Laurent 1996a) in the AL of locusts. Similar oscillations have also been observed in intracellular recordings from drosophila AL (Tanaka, Ito, and Stopfer 2009). The presence of such oscillations suggests that odor induced PN responses are correlated with more PNs spiking at the peak of the LFP than at other phases. The oscillations also provide a natural time scale to partition the PN response into smaller 50 ms epochs (the duration of one cycle at 20Hz). We measured the time to odor initiation and the duration of a continuous PN response in units of epochs. The statistics of the number and timing of PN spikes were extracted from a survey of the literature (see table 1 - Laurent 1996b; Wehr and Laurent 1996; Stopfer, Jayaraman and Laurent, 2003; Wilson and Laurent 2005). We adapted these results to design a matrix representation of PN activity. This consisted of a 900x3000 matrix of 1s and 0s [Figure 3]. Each row represented one out of 900 PNs, and each column of the matrix represented the activity of all PNs over a 1ms time interval. The parameters (and their values) used in this process (to simulate 1 second of odor delivery and a 3-second response) are listed below (note all variables are normally distributed, and values represent mean ± standard deviation unless mentioned otherwise):

To generate a population PN response, a value used to specify the percentage of active neurons was drawn from a normal distribution with mean and variance given in Table 1. This value was used as a probability threshold to decide if a given PN fires or not. For each of the 900 PNs, a uniform random number was drawn to decide whether that PN was activated by the odor. If the random value was less than the probability threshold chosen, then the neuron was activated by the odor. A value of the basal firing rate (per second) was drawn from a normal distribution with the appropriate mean and standard deviation (Table 1) and spikes equaling three times the value drawn were uniformly and randomly distributed over the 3000 time points. A value for odor induced firing rate was drawn from a normal distribution, as were the number of active epochs and the number of epochs before odor-induced activity. These three values provide information about which of the LFP oscillation cycles additional spikes needed to be added to the particular neuron's activity, as well as how many spikes were to be added in a single epoch. These spikes were then distributed in each of the "active" epochs in such a way that the spike was more likely to occur at the center of the epoch (corresponding to the peak of the LFP) than at the ends. If the neuron was not odor-activated, then it fired at its basal firing rate as described earlier.

These attributes were calculated for each of the 900 PNs to generate a complete spatiotemporal pattern describing an odor. An odor was defined by the specific PNs that were activated and the parameters drawn from the distributions quantified in Table 1. In different trials of the same odor, the PNs that were activated, as well as their parameters, remained the same. However, the exact timing of the spikes in the active epochs changed.

The timing of spikes was drawn randomly (within specified "active" epochs) for each trial. In contrast, two odors differ not only in the timing of spikes of active PNs but also in the identity of the active PNs.

Whether a PN was active or not was independent of whether other PNs were active. This reflected the multi-glomerular organization seen in locust. To mimic a fly-like glomerular organization where sister PNs fire in a correlated manner, PNs were divided in 50 groups of 6 (Note that here we simulated 300 PNs and not 900 in agreement with the number seen in the fly). The grouping reflected the glomerular architecture in Drosophila. 5 out of these 50 groups were chosen to contain active neurons. The other 4 parameters mentioned in Table 1 were then chosen for these active neurons. To simulate a new odor that was distinct from a previously described one, 1-5 of the active glomeruli in the first odor were changed randomly (See Figure 3 for an instance of a simulated odor).

### Neuron and synapse implementation

The spatiotemporal pattern that was generated using specific attributes for PN spike statistics described above was used to stimulate a layer of 50,000 KCs. We systematically varied PN-KC connections and computed the corresponding KC responses to several odors. PN-KC synapses are cholinergic (Yasuyama 1999) and were modeled as such [equations 1, 2, 3] (Destexhe, Mainen, and Sejnowski 1994; Bazhenov et al. 2001; Javier Perez-Orive et al. 2002; Turner, Bazhenov, and Laurent 2008). Each PN spike released a fixed amount of neurotransmitter T. This was used to drive post-synaptic KCs. The synaptic currents were given by:

(1) $$I_{syn}=g_{syn}\times \left[O\right]\times \left(V-E_{syn}\right)$$

Where,

(2) $$\frac{d\left[O\right]}{dt}=\alpha \times \left(1-\left[O\right]\right)\times T-\beta \times \left[O\right]$$

(3) $$T=A\times \Theta \times \left(t_{0}+t_{max}-t\right)\times \left(t-t_{0}\right)$$

In these equations the constants were: *α* = 0.94 ms^−1^, *β* = 0.18 ms^−1^, $g_{syn}=0.05\frac{mS}{cm^{2}}$, *E_syn_* = 0 mV and *t_max_* = 0.3ms Θ is the Heaviside function. [O] is the open probability of the ion channels on the KC membrane and *T* represents the amount of neurotransmitter released by a given PN. t_0_ is the time of the last spike and t_max_ is the duration for which the neurotransmitter was released. KCs were modeled as leaky integrate and fire neurons (Turner, Bazhenov, and Laurent 2008; Papadopoulou et al. 2011).

(4) $$C_{m}\frac{dV}{dt}=-g_{L}\left(V-E_{L}\right)-I_{syn}$$

Here $g_{L}=0.089\frac{\text{mS}}{{\text{cm}}^{2}},C_{m}=1\frac{\mu F}{{\text{cm}}^{2}}$ and *E_L_* = −65 mV. The KC generated a spike when *V* > *V_thresh_*. The membrane potential was reset to −65 mV at the time point immediately after the spike. We simulated an array of 50,000 such KCs that responded to a 3000ms long input from PNs.

### Classification and distance metrics

To quantify the difference between the representations of two odors by the same neuronal population we used the Hamming distance. Elements of the KC activity vector were set to 1 if that KC fired a spike during the odor presentation and zero otherwise. The Hamming distance calculates the number of bits that differ between the two vectors (For example see Figure 2). In some figures, we used a normalized version of this metric that divides twice the Hamming distance by the total number of active neurons in both vectors being compared. To illustrate this metric, consider a vector representing the activity of 100 neurons. Consider, in one scenario 10 of these neurons were active for odor A and a different set of 10 non-overlapping neurons for odor B. The Hamming distance between these odor representations would be 20. In another scenario, 20 neurons were activated for odor A and 20 non-overlapping neurons for odor B, the Hamming distance would be 40. However, in both cases the two odors were maximally different from one another, that is, they did not overlap. In contrast, the normalized Hamming distance for both cases described above would take a maximum value of 1. The normalized Hamming distance may be thought of as a measure of the degree of overlap between odor representations. If two odors stimulate strictly non-overlapping KCs the distance between the representations would be 1 regardless of the number of active KCs. This normalization was also necessary to visualize the distance between odor representations particularly when the PN-KC connections were dense (>50%). Dense connectivity regimes showed a large trial-trial variation in the number of active KCs.

In addition to using the normalized Hamming distance to visualize the distance between odor representations, we used two classification algorithms (k-medoids clustering and non-classical multidimensional scaling) to visualize and classify high dimensional KC representations of odors. In both these classification algorithms we first defined the pairwise Hamming distance between the KC vectors of all simulated odor representations. The algorithm (k-medoids clustering using MATLAB) iteratively minimizes the within cluster distance while maximizing the distance across clusters. Unlike the k-means clustering algorithm that calculates a center for each cluster as the mean of the cluster, the k-medoids algorithm treats an existing data point as the center of the cluster and measures all within-cluster distances from that point. We also performed a multidimensional scaling analysis using the mdscale function in MATLAB. The algorithm maps points from the high-dimensional KC space to a plane while preserving the pairwise distance relationship between all the data points.

### Code Accessibility

The code/software described in the paper is freely available online at http://modeldb.yale.edu/261877. The access code for the online repository is 0000. The code is also available as Supplementary Code.

## Results

In the locust, each KC receives input from nearly half of the antennal lobe PNs. This pattern of connectivity maximizes the difference between inputs to any two of the ~50,000 Kenyon cells in the mushroom body [Figure 2b] (Jortner, Farivar, and Laurent 2007). Given the large number of possible combinations of inputs to KCs, it is highly unlikely that the combination of PNs that synapse onto a given KC will be exactly the same as that which synapse onto any other KC. In contrast, if the PN-KC connection probability were 5% (seen, for example, in drosophila), the number of total possible PN combinations would be nearly 99% lower than if the PN-KC connection probability were 50%, making it more likely for two KCs to share the same inputs [Figure 2b], (Jortner, Farivar, and Laurent 2007, Jortner 2013). What advantages does this seemingly sub-optimal scheme offer? We addressed this conundrum by simulating a model KC network that received realistic PN input. Using the distance between KC odor representations, and the classification accuracy of the network, as a proxy for the ability of the animal to distinguish odors, we determined the circumstances under which different circuit connectivities confer specific advantages in odor discrimination.

### A PN identity code allows a wide range of connectivities to distinctly represent odors

If each KC sees *m* out of *n* PNs, then the maximum number of combinations would be obtained for $m=\frac{n}{2}$ [Figure 2b]. However, it is the response of KCs that is read by subsequent layers, not PN input. The KC response may be thought of as a nonlinear transformation of the summed input from the PNs. KCs act as coincidence detectors that integrate pre-synaptic input that arrives within short temporal windows of the order of ~50ms (Perez-Orive J. et al. 2004, Perez-Orive J. et al. 2002, Gruntman & Turner 2013). KCs fire only if a sufficient number of spikes fall within the integration window. Therefore, we first investigated whether the previously hypothesized (Jortner, Farivar, and Laurent 2007) optimal connection probability from PNs to KCs remains optimal in spite of the threshold imposed by the KC response and whether a lower connection probability is indeed sub-optimal.

We tested this hypothesis using a simple threshold model of KCs and determined how distinctly the KC population output represented different odors. We modeled the input to KCs as a binary vector of length 900. This captured a single snapshot of the activity of the AL circuit (Jortner 2013; Litwin-kumar et al. 2016) [Figure 2a]. In the locust AL, the duration of each cycle of the 20 Hz oscillatory local field potential provides a natural time-scale to define the duration of a snapshot. We then calculated the response of KCs to this input for different values of PN-KC connectivity. We varied the number of projections from PNs to KCs such that each KC received inputs from 5 to 95 percent of all PNs (in steps of 5 percent). We simulated different odors by randomly shuffling the PN activity vector. If the summed activity of all the PNs that were connected to the same KC exceeded a threshold, we labeled the KC as active and set its response to 1. Increasing the density of connections from PNs to KCs increased the number of active KCs for the same input vector. Changes in the sparseness of the KC output vector can lead to a change in the distance between odor representations. Our goal was to calculate the overlap between output vectors, independent of the sparseness of the representation. Therefore, for each connection probability we adjusted the response threshold of KCs such that only 10% of the 50,000 KCs simulated crossed the threshold. (Javier Perez-Orive et al. 2002; Turner, Bazhenov, and Laurent 2008). This ensured that changes in the distance between odor representations were solely due to changes in the PN-KC connectivity and not confounded by connectivity dependent changes in the sparseness of the KC response. We simulated four sets of inputs consisting of 101 PN odor representations. Within each of the four sets of simulated odors, the input vectors differed from each other by varying amounts - 5, 10, 20, 40 or 80% respectively. For example, consider the 900 PNs whose activity represented a given odor ‘A’. About 20% of these PNs would be active. Another odor ‘B’ in the input set would differ from ‘A’ by 10% if 90 of the 900 PNs changed their activity state from active to inactive or vice versa when compared with ‘A’. We then calculated the normalized Hamming distances between odor pairs belonging to each group and compared the distances obtained for different PN-KC connection probabilities. The KC population's ability to distinctly represent odors showed no dependence on the connectivity between the two regions [Figure 2c] regardless of the degree of similarity between the PN representations of odors. This counterintuitive result arises from the fact that even at low connectivity values the number of ways to choose inputs to KCs is more than a hundred orders of magnitude greater than the number of KCs in the network (Litwin-kumar et al. 2016)(see the Discussions section). Therefore, when odor distances were measured in terms of the output of KCs, both the drosophila (5% PN-KC connectivity) and the locust olfactory network (50% connectivity) were equally capable of distinguishing between similar odors.

### Inclusion of PN temporal patterning reveals the functional differences between connectivities

In response to an odor presentation, AL neurons generate a dynamic pattern that evolves reliably and over multiple time scales. This spatiotemporal patterning is thought to progressively decorrelate the representations of similar odorants (Wiechert et al. 2010) and make them more easily discriminable by follower neurons in the mushroom body. Earlier, we used a single snapshot in time to represent an odor and found that the PN-KC connectivity had little effect on the Hamming distance between KC representations of the odor. Next, we sought to determine the role of the temporal structure of odor representations in discrimination.

Odor inputs to KCs were modeled as a pattern of spikes from PNs. The statistics of spikes emulated that seen in the extant literature (see methods). We simulated trial-trial variability by jittering the spike timing within 50 ms windows. Note that in addition to this jitter, random spikes were inserted such that the mean baseline firing rate in the absence of an odor stimulus was 4 Hz. We simulated different odors by activating different groups of PNs. To visualize the dynamics of the population of PNs, we first calculated the number of spikes generated by each PN in overlapping 50 ms windows. We then projected the PN activity vector during each 50ms window onto the first three principal components. Odor representations of the PN population may be visualized as continuous trajectories in this reduced-dimensional space. When the odor stimulus was turned on, the AL response followed a trajectory from baseline (defined by low firing rates) to a ‘fixed point’ (Mazor & Laurent 2005). Once the odor stimulus was turned off, the trajectory returned to baseline, but along a different path from the one it had taken to reach the fixed-point post-odor-onset (Mazor & Laurent 2005, Stopfer et al. 2003). Multiple trials of the same odor generated trajectories that remained close to each other, while dissimilar odors were well separated in the space defined by the principal components. [Figure 3]. The input from PNs was used to drive a population of KCs. In contrast to the threshold model of KCs used in the previous section, here we modeled KCs as leaky integrate and fire neurons with integration properties that matched the responses seen in earlier studies (Javier Perez-Orive et al. 2002; J. Perez-Orive, Bazhenov, and Laurent 2004). Here too, we maintained the sparseness of KC responses across different PN-KC connection regimes by choosing progressively higher spike thresholds as the probability of connections increased. The threshold chosen ensured that only 10% of the KCs spiked in each epoch (50ms window) when the odor was present regardless of the connectivity. We chose such a threshold-based sparseness to mimic the ultimate effect of the GGN that dynamically adjusts feedback inhibition in response to the intensity of the KC response. However, for high PN-KC connectivity, (>50%), we found that the difference between inputs to different KCs was very small. Therefore, small changes in the KC threshold led to an all-or-none response and consequently a high variability across trials and a reduced ability to discriminate between odorants. Intrinsic variability in KC thresholds and differences in the strengths of PN-KC synapses can potentially reduce this variability for connectivities beyond 50%. We used a normalized Hamming distance to visualize differences across all connectivity values. In the 0-50% connectivity regime, where the number of activated KCs remained nearly the same and well-controlled by KC threshold modification, the Hamming distance matched the normalized Hamming distance except for a constant scaling factor. Including PN temporal patterning revealed some functional differences between different PN-KC connectivity regimes.

**Figure 4:**
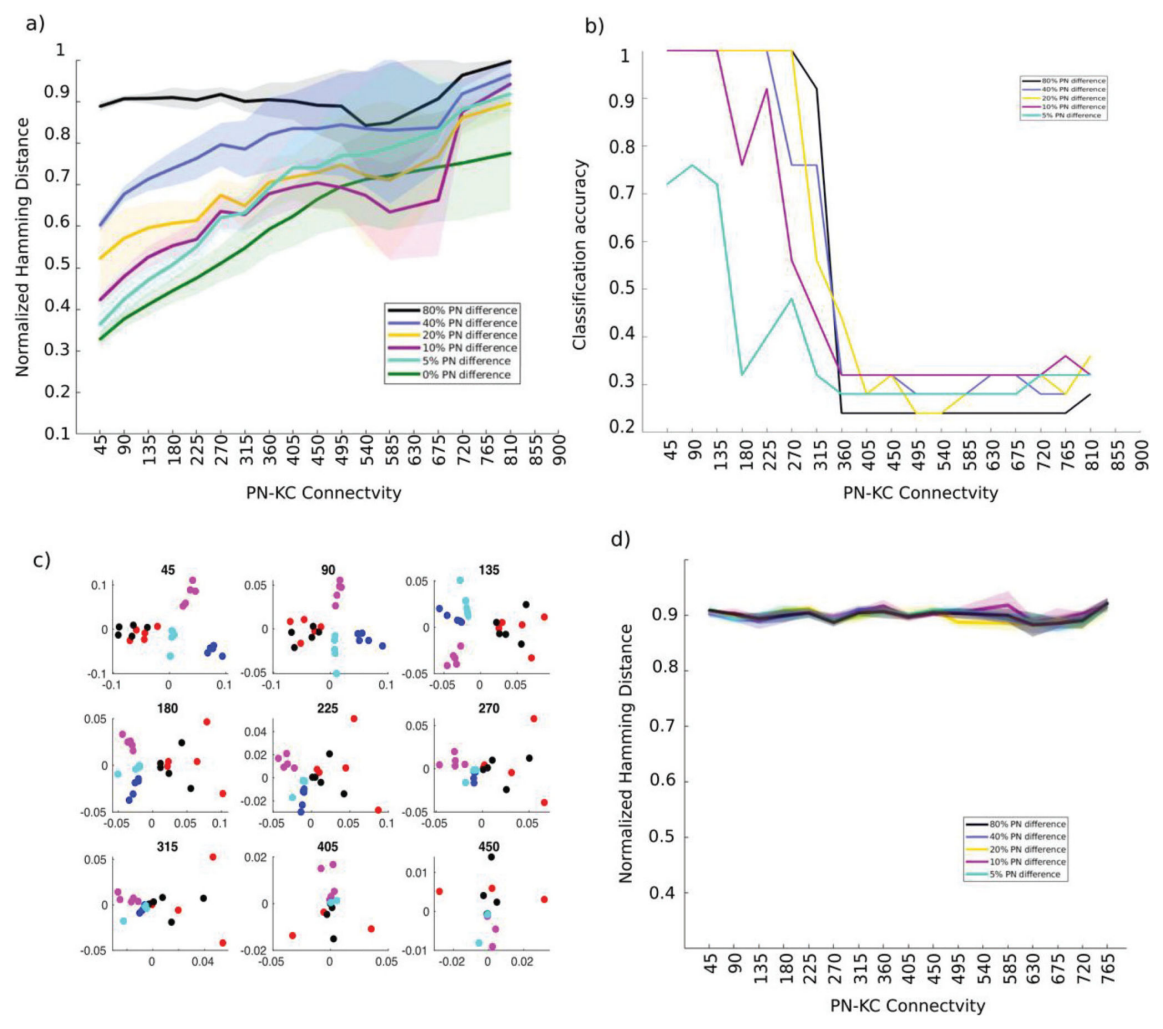
PN temporal patterning reveals the functional differences between connectivities a) Distance between odor representations. The mean (± standard deviation) normalized Hamming distance between the KC representations of odor pairs is shown as a function of the PN-KC connectivity value. Here KCs are modeled as described in Figure 3. b) Classification accuracy decreases with increasing PN-KC connectivity. A k-medoids clustering algorithm that used the distance between 25 KC activity vectors (5 trials x 5 odors) was used to categorize each vector as one of 5 odors. The percentage of correctly classified odor representations is plotted on the y-axis as a function of the connectivity of the PN-KC network. c) Odor representations become indistinguishable with increasing PN-KC connectivity. Five odors that differed from each other by 5% PN input, mapped to a plane using multidimensional scaling. Different trials of a given odor are plotted using a single color. Different odors are plotted using different colors. The PN-KC connectivity is shown in the title of each sub-plot d) Hamming distance between static odor representations. The mean (± standard deviation) normalized Hamming distance between the KC representations of odor pairs is plotted as a function of PN-KC connectivity. Here, the PN odor representation did not change in time.

KCs received inputs that represented odors with different degrees of similarity between them. We calculated the mean normalized Hamming distance between all pairs of KC activity vectors for different odors and connectivities [Figure 4a]. Our analysis began to pick out differences in the ability of the KC population with different connectivities to represent odors distinctly. The normalized Hamming distance between KC odor representations increased with increasing PN-KC connectivity for all odor distances [Figure 4a]. This implied that the representations of two different odors are more distinct in higher connectivity regimes. This could potentially allow the network to accurately associate specific odors with reward signals in downstream layers of the olfactory circuit (Cassenaer & Laurent 2012, Owald et al. 2015, Hige et al. 2015). However, an increase in Hamming distance was accompanied by a concomitant increase in the variability of the distance across odor pairs. We found a similar trend in the distance between the trials that represented the same odor (trace marked 0% difference in Figure 4a). Therefore, for high PN-KC connection densities, it seemed likely that different trials of the same odor could be incorrectly classified as distinct odors. Ideally, the network must maximize the distance between odor representations while also keeping the trial-trial variability within a range that prevents misclassification of odors. The Hamming distance metric does not take into account the variability of KC odor representation. Therefore, we used k-medoids clustering to separate the odor representations into non-overlapping groups. Our data consisted of 25 KC response vectors (5 odors x 5 trials). Each was a 50000-element long vector, where each element represented a single KC and contained either a 1 if that KC was active or 0 if it was inactive. We determined whether the trials had been grouped correctly based on their odor identity. For each set we used the percentage of correct classifications as a measure of the ability of the network to distinguish between odorants. As the PN-KC connectivity increased to nearly 45%, the number of correct classifications dropped abruptly, indicating that the distance across trials of the same odor matched or exceeded the distance between representations of different odors [Figure 4b]. Therefore, 45% PN-KC connectivity increased the distance between representations while keeping trial-trial variability within a reasonable range. This result is similar to that of (Jortner 2013) though it is based on the output of KCs over a few seconds of odor stimulation, while (Jortner 2013) based their conclusion on a single snapshot of odor input. Next we used multidimensional scaling to visualize the distribution of different odors on a plane. The algorithm mapped each 50000-dimensional KC representations of an odor trial on to a single point on this plane. For low values of PN-KC connectivity, multiple trials of the same odor preferentially remained close together. As the divergence of connections increased, the separation between the representations of different trials of a particular odor and different odors began to merge, making it difficult to correctly segregate the odors [Figure 4c, different odors are marked in different colors]. The odors plotted here differed from each other in 5% of the PNs that were stimulated.

It is possible that the differences in Hamming distance could be merely a consequence of using a specific KC model (an integrate-and-fire neuron here) compared to a nonlinear threshold neuron in earlier sections. To show that this is not the case we created odor representations in which odors differed only in the identity of PNs that they activated. All active PNs produced the same number of spikes at exactly the same points in time. In this way we continued to include all aspects of our expanded model but removed any differences in temporal structure that could be utilized differently by the different connectivity regimes. Therefore, if the usage of our new KC model that evolved in time was the cause for the functional differences that we saw, then the results of this simulation would differ from that of the previous simulations [Figure 2c] that used a threshold model. We found that the distance between odor representations in both models, the integrate and fire model and the threshold model, were independent of the degree of PN-KC connectivity when temporal features of the odor representation were eliminated (compare Figure 4d with Figure 2c).

Taken together, these results suggest that the inclusion of temporal structure in AL activity causes post-synaptic KC populations that receive a large number of inputs to respond differently from those that receive few inputs. However, there appears to be a trade-off here. Dense connectivity regimes are highly sensitive to small changes in incoming input and can incorrectly categorize noisy trials of the same odor as different odors. On the other hand, sparse connectivity regimes produce reliable representations that can be clustered correctly into different groups. However, these are likely to fail if very similar odors are introduced because the representations may not be well separated as seen from the low Hamming distance between the odor representations [Figure 4].

### Glomerular organization of the fly aids odor discrimination

Olfactory receptor neurons in insects are distributed randomly across the antennae within tiny hair like structures called sensilla. Each receptor neuron expresses a single olfactory receptor protein and possesses a receptive field tuned to a variety of odorants (Hallem and Carlson 2004, 2006). In drosophila, all the sensory neurons expressing a particular receptor type synapse onto a single glomerulus giving nearly identical input to sister PNs that receive input from that glomerulus (Kazama and Wilson 2009). While correlated PN responses can potentially improve the signal to noise ratio, this comes at a cost, namely, the dimensionality of the olfactory representation is vastly reduced. The size of the representation may be thought of as the number of independent dimensions, that is, the number of neurons that can generate uncorrelated patterns of activity. In locusts that lack this glomerular organization, the maximum number of independent dimensions is 900 (number of PNs that could potentially receive unique odor input). In drosophila this reduces dramatically since multiple neurons receive identical input from ORNs and generate a highly correlated output. The number in drosophila may be much smaller (~50, the number of glomeruli) since the output of sister PNs is nearly the same. Does the glomerular organization of the drosophila olfactory system mitigate some of the disadvantages in odor discrimination imposed by sparse PN-KC connections?

**Figure 5:**
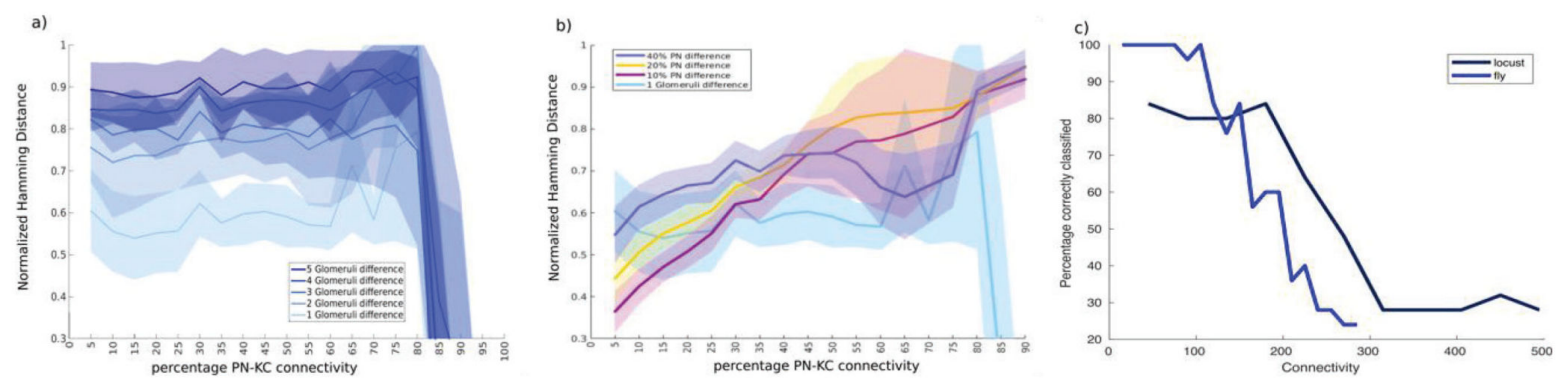
Glomerular organization of the fly aids odor discrimination (a) The mean (± standard deviation) normalized Hamming distance as a function of PN-KC connectivity in a network with glomerular structure. (b) The normalized HD of odors with a 1-glomerulus difference in a fly-like glomerular system is compared to the HD between odor representations of a system with locust-like glomerular structure. (c) Classification accuracy of odors that are different by 2 glomeruli (2% or 12 neurons in the fly-architecture) (blue trace) compared to the classification accuracy of odors that differed by 5% (45 neurons) of stimulated odors in locust. Classification accuracy is higher for the fly-like organization for low PN-KC connectivities.

To test if the inclusion of the uni-glomerular architecture seen in the fly produces any improvement in the ability of sparsely connected networks we performed simulations in which odors were defined by the glomeruli they activated. These odors differed in the number of unique glomeruli they activated rather than the number of unique PNs [Figure 5a]. These inputs were then fed to the same KC network simulated earlier. We saw that for sparse connectivity regimes the uni-glomerular organization magnified the differences in PN activity and increased the Hamming distance between KC representations of odors compared to the non-glomerular case [Figure 5b]. We then used k-medoid based clustering and classification to determine whether the fly-like architecture provided any benefits in odor classification. We compared the classification accuracy as a function of PN-KC connectivity for two cases – a system with a multi-glomerular (locust-like architecture) and one with a uni-glomerular (fly-like architecture). We found that the uni-glomerular architecture improved the classification accuracy of the network for low PN-KC connectivities compared to the multi-glomerular architecture [Figure 5c]. However, this kind of glomerularization appears to cause no change or even slightly reduce the ability of dense connectivity schemes to separate odor representations. This suggests that the glomerular organization seen in the fly does in fact improve the animal’s ability to distinguish between odors.

## Discussion

### Discrimination of purely spatial odor representations is independent of PN-KC connection density

In the locust AL, PNs generate elaborate spatiotemporal patterns in response to an odor. These patterns are read by KCs in the MB. The density of connections between PNs and KCs is such that each KC receives input from nearly one half of the PNs. A 50% probability of connections from PNs to KCs ensures that the PN inputs to KCs are maximally separated. The number of ways to pick *m* out of *n* elements is maximized when $m=\frac{n}{2}$, thus maximizing the distance between inputs to KCs (see Figure 2b and Jortner 2013). This argument assumed that this distance between inputs dropped off quickly as *m* changed from $m=\frac{n}{2}$. Therefore, in schemes that did not have close to 50% connectivity KCs did not receive sufficiently distinct inputs. We found that while the inputs were indeed maximally separated at 50% connectivity, once the summed inputs underwent a KC threshold function all connectivity regimes were equally good at separating odors. This is in line with more recent studies that show that even a 5% connection probability generates a large representation space such that even highly similar odors are mapped to distant locations (Litwin-Kumar et al. 2017). However, these observations are confined to odor representations that are static. When the temporal patterning of inputs was included, denser connectivities appeared to be significantly better at separating odor representations.

### Odor representations are variable in networks with dense connectivity

Increasing connection density comes at a price. Odorants are embedded in a noisy and changing milieu. Recognition of appetitive and aversive odorants must play out against a background of irrelevant olfactory information. Thus, the network must be tolerant to perturbations in the odor representation. This constraint introduces an upper bound on the density of connections between PNs and KCs. Our simulations demonstrated that high connectivity values led to highly variable representations of the odor by KCs as was seen from the standard deviation of the Hamming distance. Dense (80 – 95%) connectivity regimes generated representations that were 4 – 5 times more variable than representations generated by sparse connectivity schemes. The reason for this increased variability is that for dense connectivity schemes, KCs see nearly identical input from PNs. For connectivity regimes > 50%, with temporally varying PN inputs, the discriminability between KC inputs decreases with increasing connection density. The response of KCs is modulated by inhibitory feedback from the GGN. The GGN inhibits all the KCs and maintains sparseness across large variations in odor attributes by controlling the propensity of KCs to respond. In high connectivity regimes, a threshold that causes one of the KCs to fire invariably allows most KCs to fire. A small increase in threshold can lead to a condition where none of the KCs fire. Noisy changes in input statistics can thus drive the KC responses leading to large trial-trial variability. While the variability of the odor representation is maximal for connection densities in the 80-95% range, as mentioned previously even networks with connection densities in the range of 45-60% show poor classification ability when exposed to multiple trials of the same odor. This is clearly not ideal for a system attempting to represent sensory information in a stereotyped way over different trials and learn from experience.

### Temporal patterning of PN activity reveals functional differences amongst PN-KC connectivity regimes

A key insight from the simulations performed in this paper is the observation that the categorization of odors in the insect MB is dependent on an interaction between PN-KC connectivity and temporal patterning of PN input. The reason for these differences as shown earlier is due to the differing demands of connectivity regimes on the temporal coincidence of spiking and spike thresholds. Taken together, our results reiterate that temporal patterning of PN input carries information about the identity of odors (Stopfer, Jayaraman and Laurent, 2003). But more importantly, we show that this information can be utilized differently by systems with different PN-KC connectivity values. Sparse connectivity regimes utilize this in a way that allows for reduction in noise sensitivity and dense connectivity regimes use it to maximally separate between odors. Given the complexity of our sensory world, the olfactory system must balance two seemingly conflicting goals. Resolve highly similar sensory inputs and do so with considerable reliability in spite of noisy variations in the input. Our model suggests that the locust and drosophila live in different regimes of a continuum of possibilities, arriving at different solutions, perhaps driven by their own evolutionary histories. Importantly, the differences in the functions of these two circuits is only revealed when the temporal structure of the odor representation is taken into account.

## Supplementary Material

Supplementary Code Code to simulate PN and KC networks. The included .zip file contains MATLAB code used in the paper to produce PN network responses and simulated the KC network.

## Figures and Tables

**Table 1:** Statistics of PN spikes

Percentage of active neurons	(0.2 ± 0.05) × number of PNs
Basal firing rate	3.87 ± 2.23 spikes/second.
Odor induced firing rate	19.57 ± 10.67 spikes/second
Number of active epochs	8 ± 4 cycles of LFP
Number of epochs before activity	Number of LFP cycles drawn from a uniform integer distribution ranging from 1 to 20

## Significance Statement

How does the structure of a network affect its function? We address this question in the context of two olfactory systems that serve the same function, to distinguish the attributes of different odorants, but do so using markedly distinct architectures. In the locust, the probability of connections between projection neurons and Kenyon cells - a layer downstream - is nearly 50%. In contrast, this number is merely 5% in drosophila. We developed computational models of these networks to understand the relative advantages of each connectivity. Our analysis reveals that the two systems exist along a continuum of possibilities that balance two conflicting goals – separating the representations of similar odors while grouping together noisy variants of the same odor.
